# Design and Validation of a Modular Control Platform for a Voltage Source Inverter

**DOI:** 10.1016/j.ohx.2022.e00390

**Published:** 2022-12-29

**Authors:** Hernan Lezcano, Jorge Rodas, Julio Pacher, Magno Ayala, Carlos Romero

**Affiliations:** Laboratory of Power and Control Systems (LSPyC), Facultad de Ingeniería, Universidad Nacional de Asunción, Luque 2060, Paraguay

**Keywords:** DC/AC converter, Grid-connected converters, Power electronics, Printed circuit board design, Renewable energy, Signal processor, Three-phase inverter

## Abstract

Integrating renewable energies, such as wind or photovoltaic, requires an electronic power converter, the three-phase Voltage Source Inverter (VSI) the most common for such function. This paper presents a modular design of signal acquisition and control hardware (current and voltage) for the commercial SEMIKRON SKS35F VSI converter and a Texas Instruments TMS320F28335 DSP. Consequently, the proposed modular and open-source design allows its application in control systems of a VSI converter for isolated or grid-connected systems, applied to power generation based on renewable sources. The proposed scheme allows for a personalised design since it uses an open architecture to implement its own control algorithms that allow it to adapt to the application’s particular needs, unlike closed-architecture commercial equipment. Detailed electronic printed circuit board designs for implementation are shown on paper. Finally, the experimental tests’ results that validate the proposed design’s correct functioning are presented.

Specifications table.

**Table:** 

**Hardware name**	*Modular Control Platform for a Voltage Source Inverter*
**Subject area**	• *Electronic engineering* • *Power electronics* • *Power converters*
**Hardware type**	• *Signal measurements and sensors* • *Power electronics* • *Electrical engineering*
**Closest commercial analog**	*The closest commercial equivalent would be three-phase power inverters. The proposed design can replace such commercial products, with the advantage of offering an open and modular architecture for custom designs.*
**Open source license**	GNU General Public License (GNU GPL v3)
**Cost of hardware**	US$ 560
**Source file repository**	*Source files repository (**OSF**) write the DOI URL here. http://doi.org/10.17605/OSF.IO/5DG6X*

## 1 Hardware in context

The DC/AC conversion system was designed for alternative generation systems, such as solar photovoltaic (PV) or wind systems, taking advantage of the energy stored in a bank of batteries or capacitors. The hardware designed to control the Voltage Source Inverter (VSI) type converter includes the measurement and signal conditioning circuits used to implement the control algorithms. The designed system allows its use in a general way in any DC/AC power conversion system, and particularly for this paper, its use in applications based on renewable energies is shown. The designed hardware allows a customised implementation based on the particular characteristics of the generation system in use since the design is of open, modular and scalable architecture, as well as a controller based on a Digital Signal Processor (DSP) with an algorithm written in C language that allows quick changes of control algorithms for your optimization process. The AC/DC voltage measurement circuits and the AC/DC current measurement circuits have been designed with an output voltage range compatible with the input levels of the A/D converters of the selected DSP. These measurement and conditioning circuits have a modular structure independent from the main board to facilitate adaptability to various applications and facilitate corrective maintenance and replacement of damaged parts. The system has circuits to adapt the voltage levels necessary to trigger the Insulated Gate Bipolar Transistors (IGBTs) of the VSI. Finally, the main motherboard interfaces each acquisition module with the DSP controller.

## 2 Hardware description

### 2.1 General scheme of the experimental platform

**Fig. 1:**
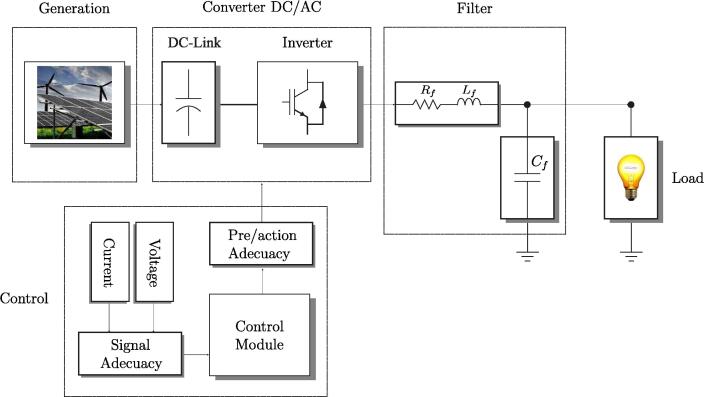
Scheme of the proposed DC/AC converter system.

The DC/AC power conversion scheme is shown in Fig. 1, in which the first stage is made up of an energy storage source based on renewable generation sources [1], [2]. The DC/AC converter stage converts the direct voltage stored in batteries to a three-phase alternating output voltage [3]. For this purpose, the Semikron SKS 35F B6U [4] converter is used. The control stage consists of voltage and current measurement boards and a DSP Control module, where the latter is in charge of generating the switching signals for the inverter based on the implemented control algorithm and measured input signals; the controller is the Texas Instruments TMS320F28335 DSP [5]. Finally, outputs of the DC/AC converter pass through an LC filter that mitigates produced harmonics by the switching nature of the IGBTs, smoothing the output in such a way as to obtain the fundamental signal of 50 Hz.

### 2.2 Design and assembly of the main motherboard

**Fig. 2:**
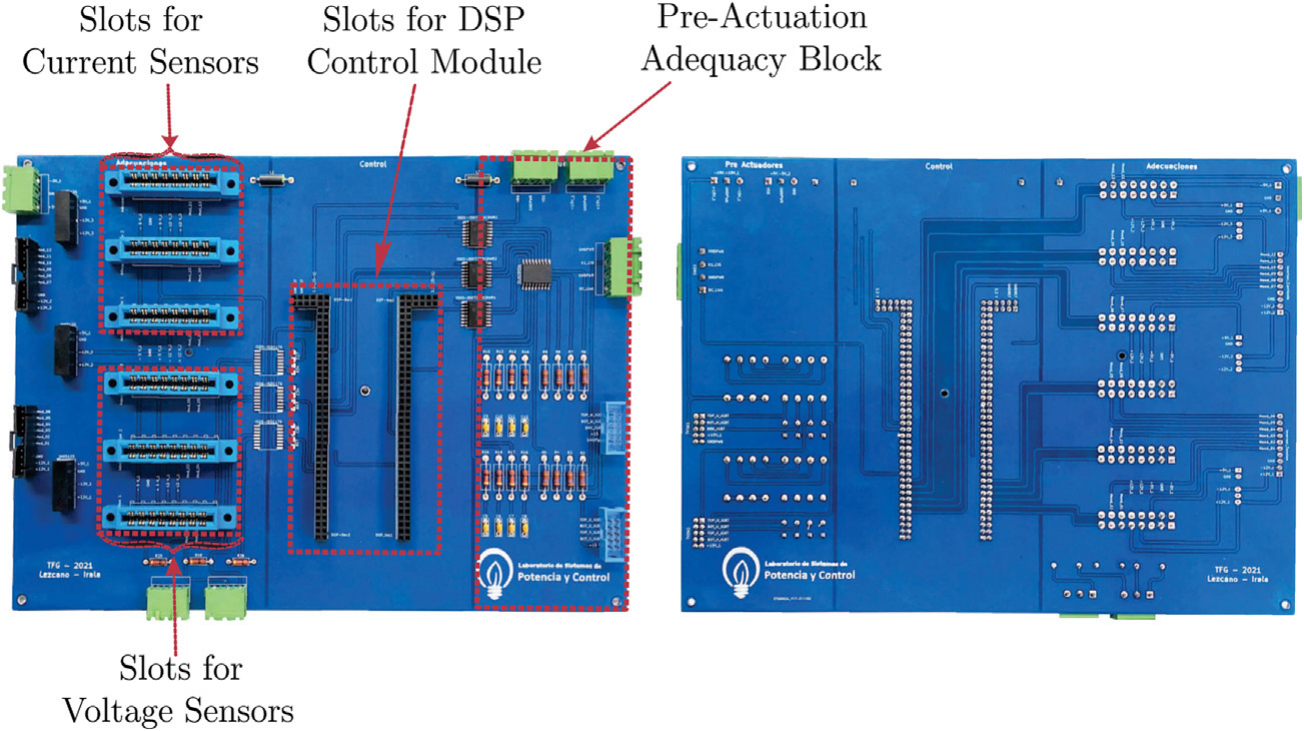
Final assembly of the Printed Circuit Boards (PCBs) of the designed circuits.

Fig. 2 shows the designed main motherboard. This motherboard has six slots where the voltage and current conditioning circuits can be inserted indistinctly since the slots on the main board and the conditioning circuits have identical power and signal connections. The main motherboard fulfils the function of bringing together the other modular plates designed and mentioned above.

### 2.3 Voltage measurement and conditioning circuit

**Fig. 3:**
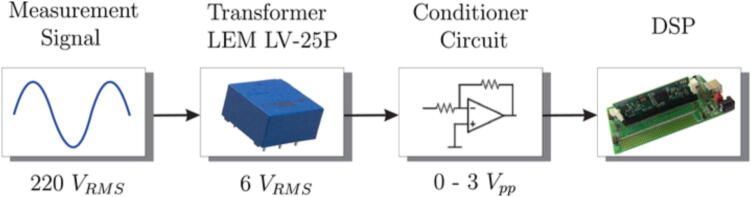
Voltage measurement schematic and conditioning circuit.

**Fig. 4:**
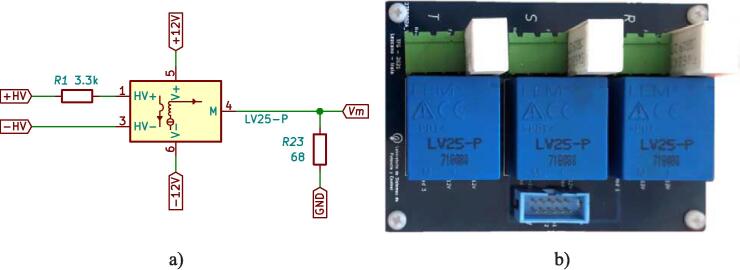
a) LEM voltage transformation scheme, b) Voltage transformation plate.

In the measurement schematic shown in Fig. 3, the signal to be measured, the sensor measurement block, the conditioning block and finally, the DSP control module can be seen. The measurement and conditioning blocks are hereinafter referred to as the measurement and conditioning circuit. This consists of three stages; the first stage is responsible for detecting the analogue signal of interest and reducing it to the DSP module’s working range, to a 3 $V_{\mathrm{pp}}$. For this, the LEM voltage sensor is used, and considering the desired input, output voltage range y the sensor specifications [6], a resistive value of $R_{1}=68$ kΩ is chosen to obtain an input current of the LEM voltage sensor of 5.5mA, with a conversion ratio equal to 2.5, a current $I_{m}$= 13.9 mA is obtained at its output. Through the resistor $R_{23}$, current $I_{m}$ is transformed into the desired voltage $V_{m}$, as shown in Fig. 4a. Resistor $R_{23}$ sets the gain of the LEM LV25-P sensor, as expressed by (1). Fig. 4b shows the plate where the LV25-P voltage transformers are mounted. This board is modular and mounts independently of the conditioning circuitry on the main baseboard.

(1) $$V_{m}=R_{23}\;I_{m}\text{.}$$

**Fig. 5:**
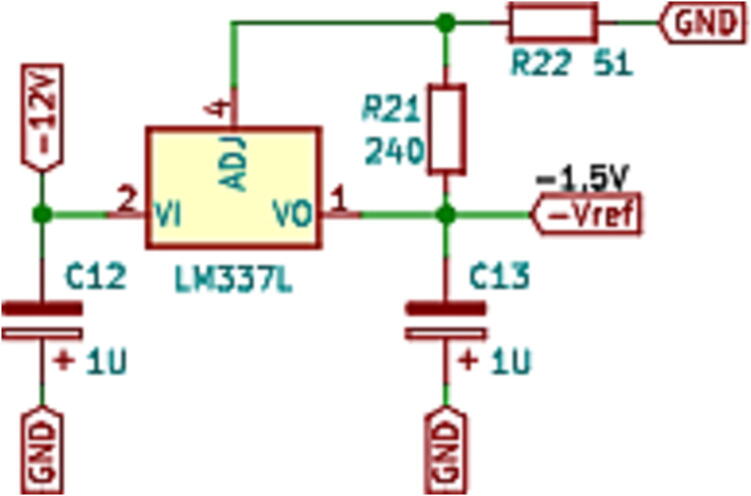
Offset generation circuit.

The second stage creates a voltage reference value of -1.5 V necessary for the offset level required by the control module. At the input of this stage, the LM337L is used, which is a negative output linear regulator. Fig. 5 shows the offset level generation circuit. The output voltage (in Volts) is set based on the selection of resistors (in Ω) $R_{21}$ and $R_{22}$, as shown in (2).

(2) $$V_{\mathrm{ref}}=V^{*}\left(1+\frac{R_{22}}{R_{21}}\right)\text{.}$$

**Fig. 6:**
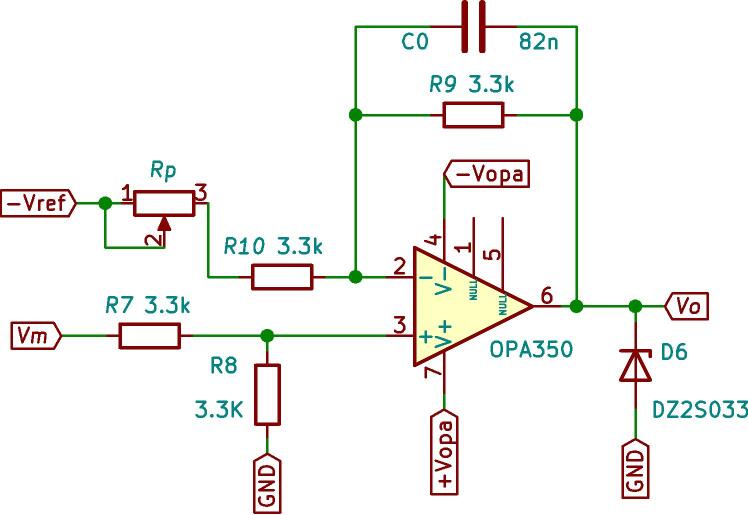
Electrical diagram of the subtracting circuit.

The selected voltage $V^{*}=-1.25$ V and resistive values for implementation are: $R_{22}=51\Omega ,R_{21}=240\Omega$. The third stage is responsible for assembling the voltage of amplitude 3 $V_{\mathrm{pp}}$ obtained at the output of the first stage (LEM LV-25P) on the continuous voltage of 1.5 V obtained in the second stage. It uses a subtracter circuit, as shown in Fig. 6. For the design of this stage, operational amplifier OPA350UA [7] is used. To obtain a unity gain, the resistors were taken to have the same value, *R* = 3.3 kΩ. Eq. (3) shows the final expression of the output voltage $V_{o}$:

(3) $$V_{0}=V_{m}-V_{\mathrm{ref}}\text{.}$$

being $V_{m}$ the output of the stage and the voltage reference $V_{\mathrm{ref}}=-1.5$ V.

**Fig. 7:**
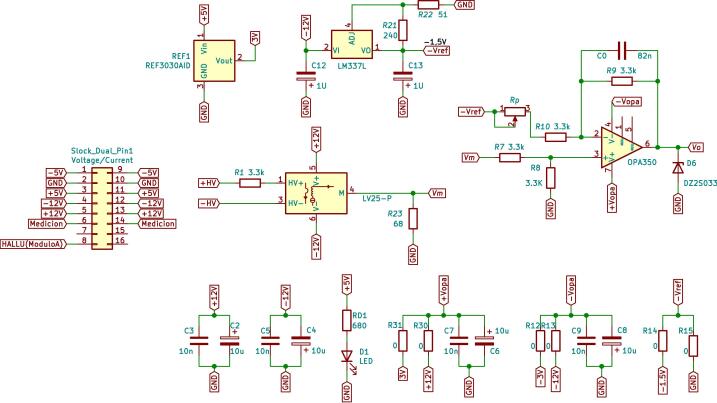
Voltage measurement schematic and conditioning circuit.

**Fig. 8:**
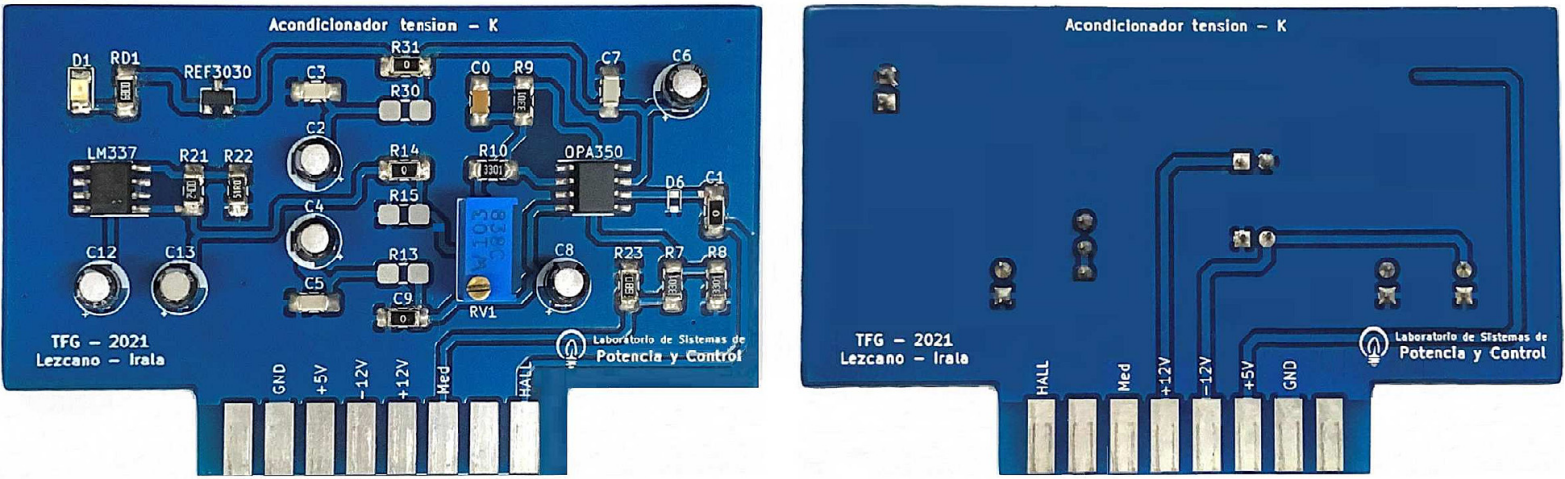
Voltage measurement and conditioning plate.

Fig. 7 shows the schematic circuit design corresponding to the conditioning of the voltage signal measurements, whose output will be connected to the A/D converter of the control board Texas Instruments DSP TMS320F28335. Fig. 8 presents the designed, assembled and calibrated voltage adjustment plate.

### 2.4 Current measurement and conditioning circuit

**Fig. 9:**
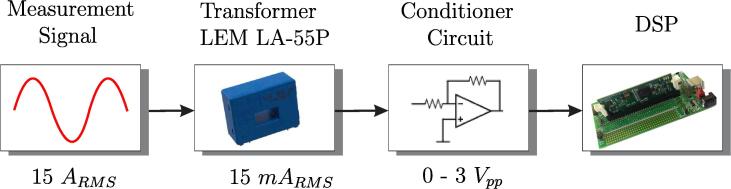
Schematic of the current conditioning circuit.

**Fig. 10:**
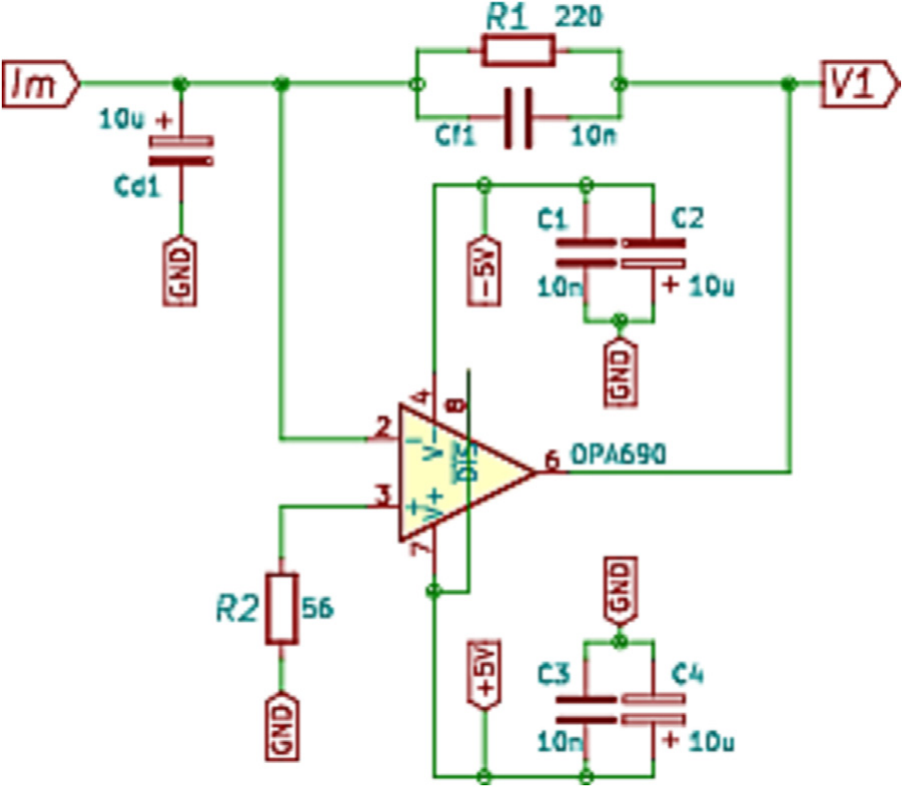
Electrical schematic of the trans-impedance amplifier.

Similarly to the previous case, the current measurement scheme and conditioning circuit consist of three stages, as shown in Fig. 9. The first stage is responsible for obtaining a voltage of 3 $V_{\mathrm{pp}}$ proportional to the output of the current sensor LEM LA 55-P [8]. For this reason, a trans-impedance amplifier is used, as shown in Fig. 10 [9]. Resistor $R_{1}$ sets the gain for this stage.

(4) $$V_{1}=R_{1}I_{m}\text{.}$$

The trans-impedance converter was designed in such a way to obtain an output voltage of 3 $V_{\mathrm{pp}}$, for a maximum input current of 14.5 $A_{\mathrm{rms}}$. The circuit gain is set as $R_{1}=220$ Ω. The Texas Instruments OPA690ID operational amplifier was used for implementation, which operates with a supply of ±5 V.

**Fig. 11:**
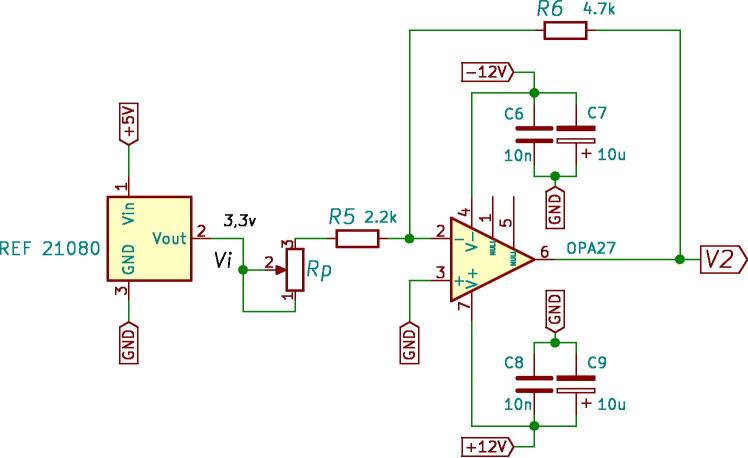
Wiring diagram of -1.5 V reference generator circuit.

Subsequently, the next stage generates a reference of 1.5 V, used for an offset voltage. At the input of this stage, a Texas Instruments REF 21080 voltage regulator is used, which generates a high-precision reference voltage of 3.3 V with a supply of 5 V [10]. This voltage enters the amplifier as shown in the circuit of Fig. 11. The output voltage of the offset circuit is given by (5).

(5) $$V_{2}=-\frac{R_{6}}{R_{5}+R_{p}}V_{i}\text{.}$$

**Fig. 12:**
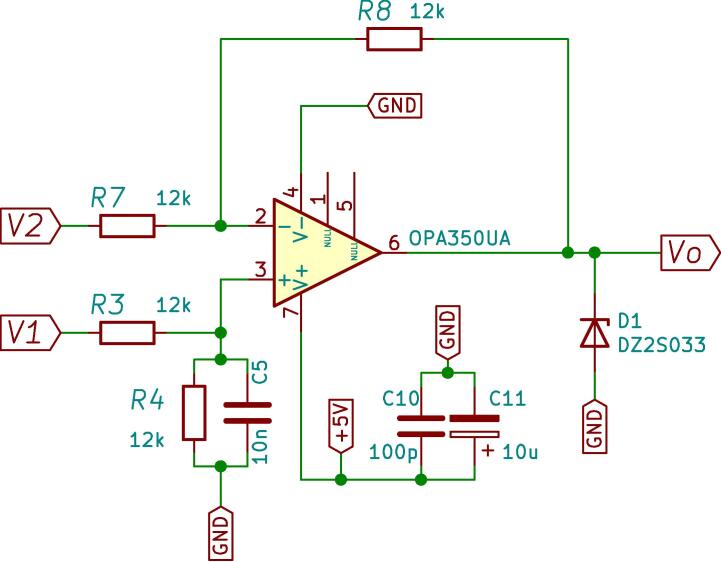
An electrical schematic of the subtracting circuit.

**Fig. 13:**
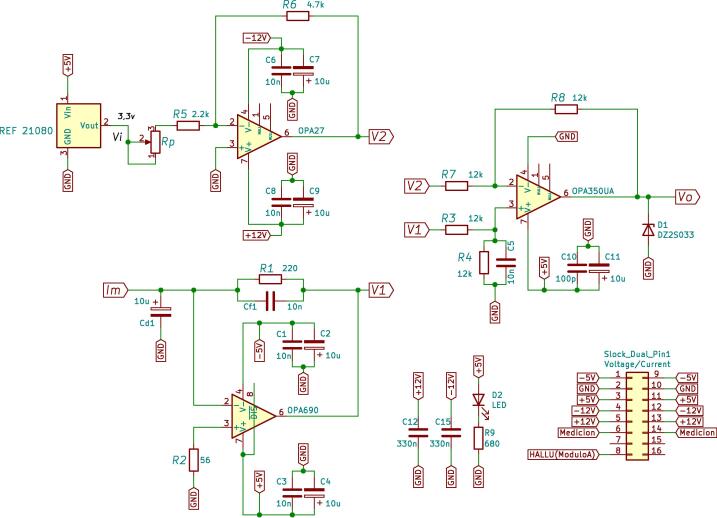
Schematic of current measurement and conditioning circuit.

The resistive values used for the design are $R_{5}$ = 2.2 kΩ, $R_{6}$ = 4.7 kΩ, where *Rp* is a potentiometer of 10 kΩ used to adjust the gain. The potentiometer is considered for fine-adjusting the output voltage to the value of -1.5 V. Then, the output voltage from the trans-impedance amplifier of the first stage is combined with the offset voltage from the previous stage. This uses a voltage subtraction circuit based on the OPA350 operational amplifier, observed in Fig. 12. Using the same values for all resistors, 12 kΩ, the output voltage $V_{0}$ is given by:

(6) $$V_{0}=V_{1}-V_{2}\text{.}$$

being $V_{1}$the output of first stage, $V_{2}$ the -1.5V reference and $V_{0}$ represents the conditioned signal. Fig. 13 shows the circuit schematic corresponding to the current adjustment stage used to condition the signals of the Hall-effect current sensors.

**Fig. 14:**
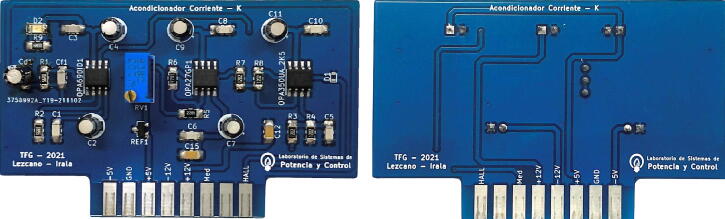
Current measurement circuit board.

**Fig. 15:**
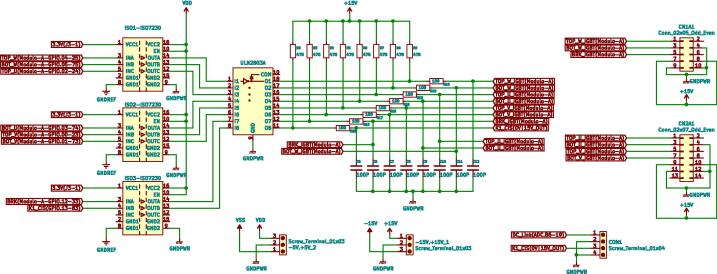
An electrical schematic of the PWM pre-actuation circuit.

Capacitance $C_{f1}$ allows the designer to compensate for the effect of the high-frequency noise introduced to the converter, making the position of the pole in a closed loop is defined by the following design equation:

(7) $$\frac{1}{2\pi R_{f}C_{f}}=\sqrt{\frac{\mathrm{GBP}}{4\pi R_{1}C_{d1}}},$$

and provides a bandwidth ($f_{-3\mathrm{dB}}$) approximately equal to:

(8) $$f_{-3\mathrm{dB}}\approx \sqrt{\frac{\mathrm{GBP}}{4\pi R_{1}C_{d1}}},$$

where *GBP* represents the gain-bandwidth product of the operational amplifier OPA 690. Taking into account the design Eqs. (7), (8)), and considering a desired output voltage of ±1.5 V, for an input current range of ±15 mA. Once the value of resistor $R_{1}$ has been determined, and knowing the bandwidth requirements, it is possible to determine the values of implicit capacitors in the design $C_{d1}=4.7$ μF and $C_{f1}=10$ ηF, respectively. Fig. 14 shows the current adaptation plate designed, assembled and calibrated. (See Fig. 15).

### 2.5 Pulse width modulation trigger signal adaptation stage

**Fig. 16:**
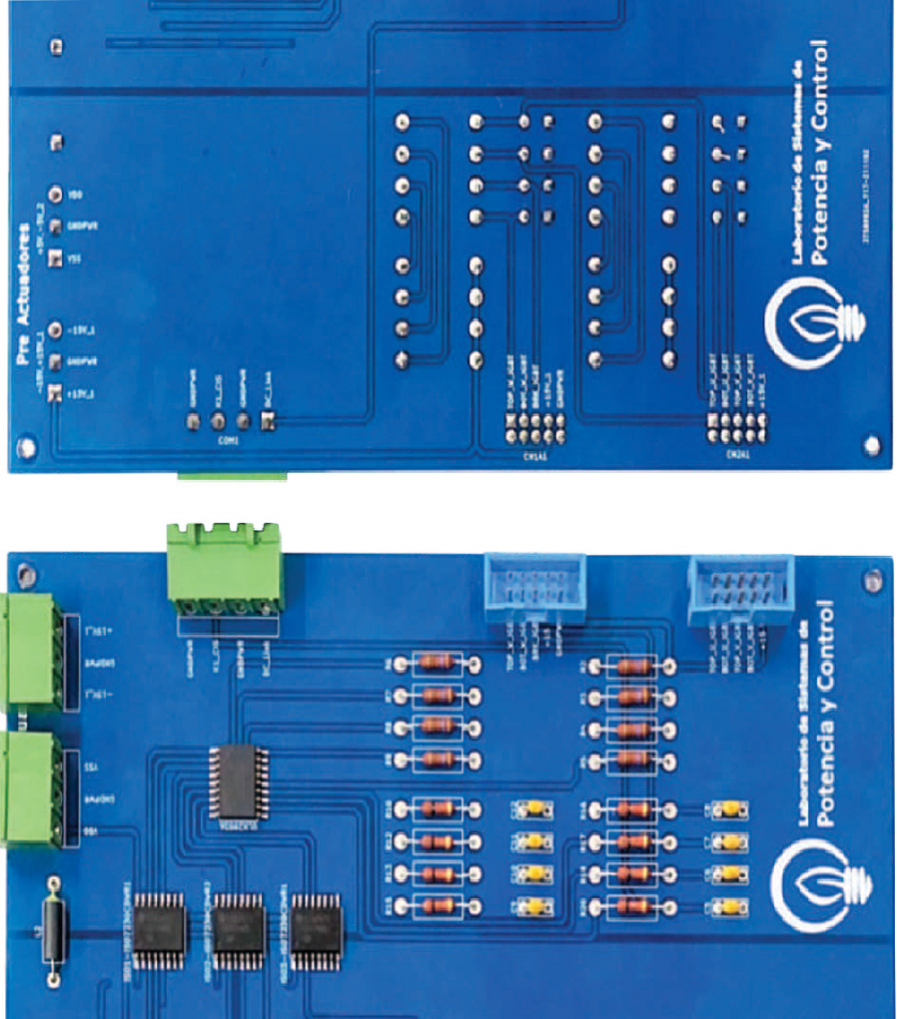
Mounted PWM pre-actuation matching block.

The proposed design meets the system’s needs in terms of bandwidth and protection. Pulse Width Modulation (PWM) pre-actuation stage is a subsystem of the coupling circuit, whose function is to convert voltage levels of digital output ports of the DSP control module, whose logic levels vary between 0 and 3.3 V, to voltage values of 0 and 15 V compatible with the operating levels of VSI SKS 35 F. In addition, this stage protects the peripherals of the DSP through a galvanic isolation network implemented using Texas Instruments ISO7240CDW series isolators. (See Fig. 16).

### 2.6 LC-based low-pass filter

**Fig. 17:**
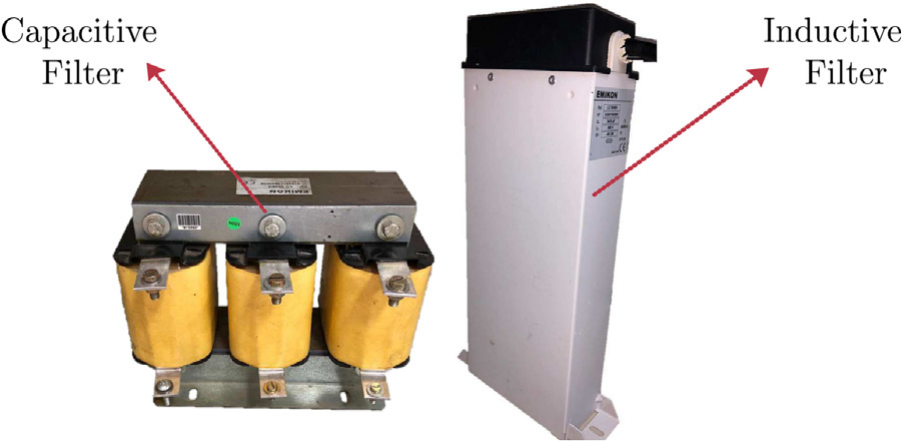
Photo of the three-phase inductance and capacitance used in the LC filter.

Then, the VSI’s output voltage must be filtered to obtain a sinusoidal output signal with the same grid’s voltage and frequency characteristics. For this purpose, an LC- Low-Pass Filter (LPF) is used, which is implemented through a 1 mH three-phase inductor and a 33 μF three-phase capacitor in delta connection. A cutoff frequency of 505 Hz is used for the values of L and C. Fig. 17 shows a photo of the elements used for the LC filter.

## 3 Design files summary

.

**Table:** 

**Design filename**	**File type**	**Open source license**	**Location of the file**
*Current Sensor*	*PCB file and Gerber, Kicad*	*GNU GPL v3*	*https://osf.io/5dg6x/*
*Current Sensor schematic.pdf*	*Current Sensor schematic file, pdf*	*GNU GPL v3*	*https://osf.io/5dg6x/*
*List of Components and prices.xlsx*	*List of Components and prices file, xlsx*	*GNU GPL v3*	*https://osf.io/5dg6x/*
*Main motherboard*	*PCB file and Gerber, Kicad*	*GNU GPL v3*	*https://osf.io/5dg6x/*
*Main motherboard.pdf*	*Main motherboard schematic file, pdf*	*GNU GPL v3*	*https://osf.io/5dg6x/*
*Software*	*Software file, Code Composer Studio*	*GNU GPL v3*	*https://osf.io/5dg6x/*
*Voltage Sensor*	*PCB file and Gerber, Kicad*	*GNU GPL v3*	*https://osf.io/5dg6x/*
*Voltage Sensor schematic.pdf*	*Voltage Sensor schematic file, pdf*	*GNU GPL v3*	*https://osf.io/5dg6x/*

• *Current Sensor: Contains the Kicad project files for the current sensor PCB and the current sensor Gerber file to produce the PCB.*
• *Current Sensor schematic.pdf: Schematic or electronic diagram of current sensor components.*
• *List of Components and prices.xlsx: Schematic or electronic diagram of current sensor components.*
• *Main motherboard: Contains the Kicad project files for the main motherboard PCB and the main motherboard Gerber file to produce the PCB.*
• *Main motherboard.pdf: Schematic or electronic diagram of main motherboard components.*
• *Software: contains programming algorithm code.*
• *Voltage Sensor: Contains the Kicad project files for the Voltage sensor PCB and the voltage sensor Gerber file to produce the PCB.*
• *Voltage Sensor schematic.pdf: Schematic or electronic diagram of voltage sensor components.*

## 4 Bill of materials summary

.

**Table:** 

**Motherboard circuit**						
**Designator**	**Component**	**Number**	**Cost per unit (US**$**)**	**Total cost (US**$**)**	**Source of materials**	**Material type**
C5, C6, C7, C8, C9, C10, C11, C12	100p Capacitor Through-Hole Technology (THT)	8	0.095	0.76	Mouser Electronics	Electronic
C17, C20, C34	100p Capacitor Surface Mount Device (SMD)	3	0.2	0.6	Mouser Electronics	Electronic
L1, L2	10μ Core_Ferrite THT	2	0.2	0.4	Mouser Electronics	Electronic
R2, R3, R4, R5, R6, R7, R8, R9	470 Resistor THT	8	0.147	1.176	Mouser Electronics	Electrical
R10, R12, R13, R15, R16, R17, R18, R20	100 Resistor THT	8	0.2	1.6	Mouser Electronics	Electrical
R24, R27, R47	100 Resistor SMD	3	0.605	1.815	Mouser Electronics	Electrical
R25, R36, R48	100 Resistor THT	3	0.408	1.224	Mouser Electronics	Electrical
U1	ULN2803A SOIC-18	1	4.69	4.69	Mouser Electronics	Electronic
ISO1, ISO2,ISO3	ISO7240 SOIC-16	3	4.69	14.07	Mouser Electronics	Electronic
ISO4, ISO5,ISO6	ISO1176 SOIC-16	3	4.69	14.07	Mouser Electronics	Electronic
PS1, PS2, PS3	IH0512S	3	7.44	22.32	Mouser Electronics	Electronic
Socket_Current	Conn_02x08	3	5	15	Mouser Electronics	Electrical
Socket_Voltage	Conn_02x08	3	5	15	Mouser Electronics	Electrical
CN1A1	Conn_02x05	1	0.35	0.35	Mouser Electronics	Electrical
CN2A1	Conn_02x07	1	0.35	0.35	Mouser Electronics	Electrical
Aliments	Screw Terminal_01x03	5	0.2	1	Mouser Electronics	Electrical
CON1	Screw Terminal_01x04	5	0.2	0.2	Mouser Electronics	Electrical
DSP_Der1, DSP_Izq1	Conn_02x04	2	0.2	0.6	Mouser Electronics	Electrical
DSP_Der2, DSP_Izq2	Conn_02x38	2	0.7	1.4	Mouser Electronics	Electrical

**Voltage measurement circuit**						

**Designator**	**Component**	**Number**	**Cost per unit (US**$**)**	**Total cost (US**$**)**	**Source of materials**	**Material type**

C0	82n Capacitor SMD	1	0.2	0.2	Mouser Electronics	Electronic
C1, C3, C5, C7, C9	10n Capacitor SMD	5	0.2	1	Mouser Electronics	Electronic
C2, C4, C6, C8	10μ Capacitor THT	4	0.15	0.6	Mouser Electronics	Electronic
C12, C13	1μ Capacitor THT	2	0.15	0.3	Mouser Electronics	Electronic
D1	Led SMD	1	0.45	0.45	Mouser Electronics	Electronic
D6	Zener SMD	1	0.45	0.45	Mouser Electronics	Electronic
R7, R8, R9, R10	3.3 K Resistor SMD	4	0.147	0.588	Mouser Electronics	Electrical
R21	51 Resistor SMD	1	0.147	0.147	Mouser Electronics	Electrical
R22, R23	240 Resistor SMD	2	0.147	0.294	Mouser Electronics	Electrical
R13, R14, R15, R30, R31	0 Resistor SMD	5	0.147	0.735	Mouser Electronics	Electrical
RD1	250 Resistor SMD	1	0.147	0.147	Mouser Electronics	Electrical
RV1	10 K Potentiometer THT	1	2.5	2.5	Mouser Electronics	Electrical
U1	LM337L	1	2.92	2.92	Mouser Electronics	Electronic
OPA350UA	OPA350UA SOIC-8	1	3.13	3.13	Mouser Electronics	Electronic
REF1	REF3030AID SOIC-8	1	1.68	1.68	Mouser Electronics	Electronic
Slock_Dual_Pin1	Connector THT	1	2.92	2.92	Mouser Electronics	Electrical
Voltage Sensor	LEM LV-25P	3	76.46	229.38	Mouser Electronics	Electrical

**Current measurement circuit**						

**Designator**	**Component**	**Number**	**Cost per unit (US**$**)**	**Total cost (US**$**)**	**Source of materials**	**Material type**

C5	10μ Capacitor SMD	1	0.2	0.2	Mouser Electronics	Electronic
C1, C3, C6, C8, Cf1	10n Capacitor SMD	5	0.2	1	Mouser Electronics	Electronic
C2, C4, C7, C9, C11	10μ Capacitor THT	5	0.15	0.75	Mouser Electronics	Electronic
C10	100p Capacitor SMD	1	0.2	0.2	Mouser Electronics	Electronic
C12, C15	330n Capacitor SMD	2	0.2	0.4	Mouser Electronics	Electronic
Cd1	4.7μ Capacitor SMD	1	0.2	0.2	Mouser Electronics	Electronic
D2	Led SMD	1	0.45	0.45	Mouser Electronics	Electronic
D1	Zener SMD	1	0.45	0.45	Mouser Electronics	Electronic
R1, R2	56 Resistor SMD	2	0.147	0.294	Mouser Electronics	Electrical
R5	2.2 K Resistor SMD	1	0.147	0.147	Mouser Electronics	Electrical
R6	4.7 K Resistor SMD	1	0.147	0.147	Mouser Electronics	Electrical
R3, R4, R7, R8	12 K Resistor SMD	4	0.147	0.588	Mouser Electronics	Electrical
R9	680 Resistor SMD	1	0.147	0.147	Mouser Electronics	Electrical
RV1	10 K Potentiometer THT	1	2.5	2.5	Mouser Electronics	Electrical
OPA27GP1	OPA27GP1 SOIC-8	1	4.27	4.27	Mouser Electronics	Electronic
OPA350UA	OPA350UA SOIC-8	1	3.13	3.13	Mouser Electronics	Electronic
OPA690ID	OPA690ID SOIC-8	1	3.13	3.13	Mouser Electronics	Electronic
REF1	ISL21080 SOT-23	1	2.06	2.06	Mouser Electronics	Electronic
Slock_Dual_Pin1	Connector THT	1	2.92	2.92	Mouser Electronics	Electrical
Current Sensor	LEM LA-55P	3	29.44	88.32	Mouser Electronics	Electrical

## 5 Build instructions

The designed PCB of all employed Integrated Circuits (ICs) on the conditioning board are of surface mount packages. Several components were not implemented in their libraries, so they were designed. The schematic diagram and PCB layout were developed using the KiCad application.

It should be noted that the main board was designed in two layers to reduce the dimension of the resulting board and use the modular design concept, leaving this main board as the motherboard for the adaptation boards. Different ground planes were placed on both sides for digital and analogue grounds. Both ground planes are connected with ferrite to reduce external noise’s influence. The advantage of the implemented design is its modularity, which facilitates maintenance and repair work due to the rapid exchange of defective or damaged parts.

The main board has a dimension of 250 mm × 180 mm. The PCBs were elaborated in JLCPCB factory: PCB Prototype & PCB Manufacture Manufacturer by sending Gerbers files generated in KiCad software.

**Fig. 18:**
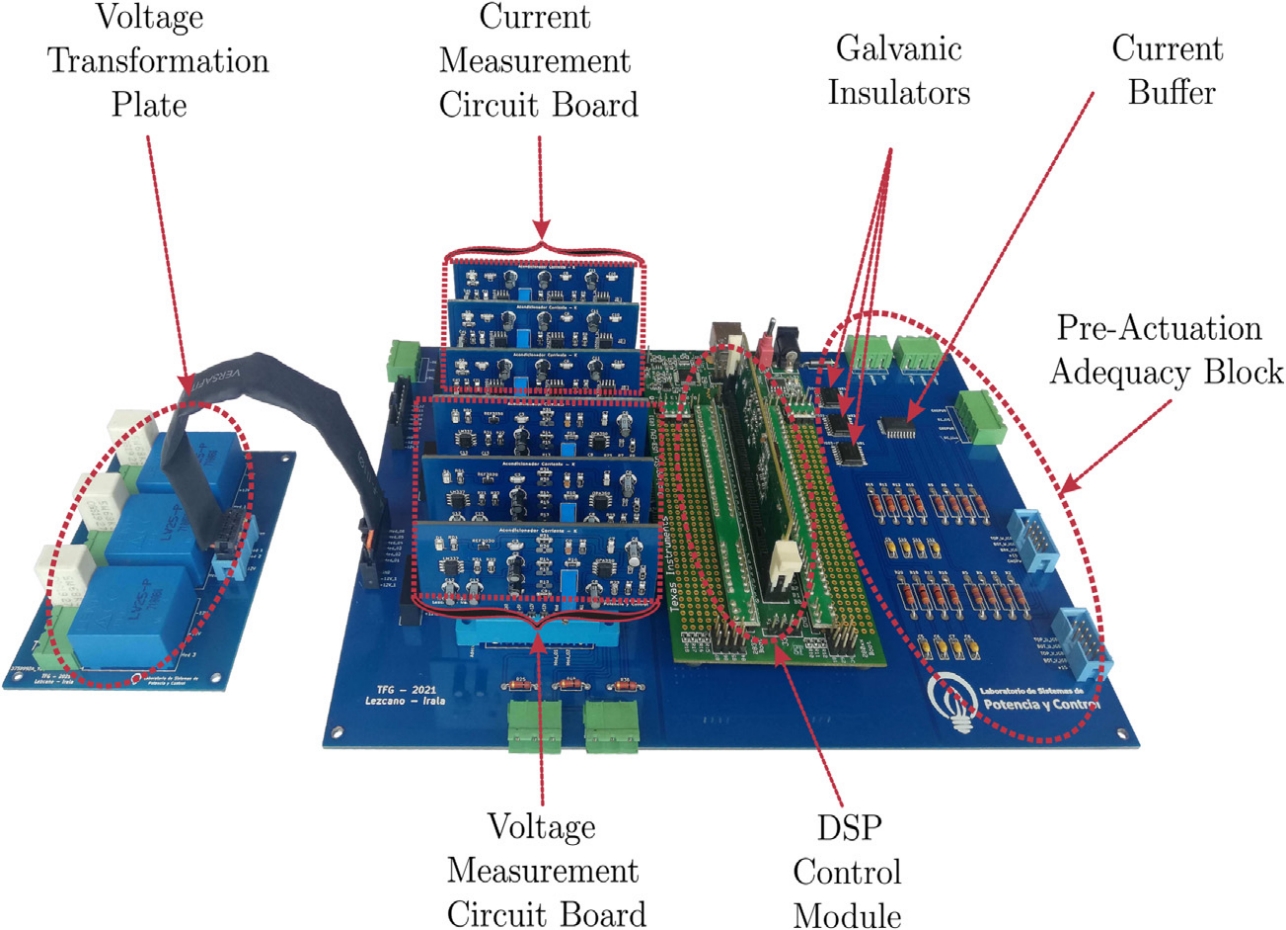
Mounted main board.

All paths on the board were subjected to continuity tests to verify that there were no short circuits between paths. The surface mount components were soldered with a soldering station and were the first to be assembled, followed by finishing with the through-hole components. Component names are clearly labelled on the PCB silkscreen and correspond to the names in the bill of materials (column designator). Fig. 18 shows the main board with other mounted and embedded boards.

## 6 Operation instructions

In what follows, step-by-step operational instructions for operating the hardware are presented.

1. Check on the dashboard that safety switch is in ON position and the emergency stop button is in correct position. This is a safety measure to ensure better equipment operation and reduces the chances of human error or accidents.
2. Activate the contactor, check that the power supplies is on and verify that pre-charge activation timer deactivates after 4 s.
3. Connect the computer with USB cable.
4. Start the programming interface of the DSP Control Module (Code Composer Studio).
5. Compile pseudo-code of the control algorithm.
6. Perform experiments.
7. Flip the safety switch back to OFF position once you are done, and close the interface.**Safety Notice**: High voltage is handled at the input of the voltage transformation board corresponding to the left side of the board. Therefore, precautions should be taken to prevent users from accidental shocks. Do not touch any of the components connected to the high-voltage terminals.

## 7 Validation and characterisation

For a correct operation of the control algorithms, it is necessary to carry out a calibration process of the signal conditioning circuits and sensors working in the appropriate range. In the calibration process, maximum values supported by the DSP must be considered for a correct and precise D/A conversion.

### 7.1 Voltage measurement circuit calibration

The voltage measurement and conditioning circuit was designed to measure voltage levels of the electrical grid 220/380 $V_{\mathrm{rms}}$. The results obtained from the circuit calibration process are summarised in Table 1.

**Table 1:** Characterisation of voltage sensors.

Sensor	Input voltage	Output voltage	Output/Input ratio
Sensor 1	220 V	2.282 V	10.37272727x$10^{-3}$
Sensor 2	220 V	2.281 V	10.36818182x$10^{-3}$
Sensor 3	220 V	2.248 V	10.21818182x$10^{-3}$

**Fig. 19:**
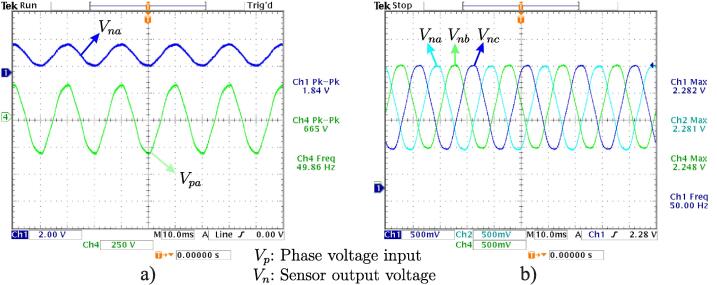
a) Comparison of 220 V input and sensor output signal, b) Three-phase output signals measured with voltage sensors.

Fig. 19a shows 220 V input voltage signals and 3 V output signal, obtained after the calibration process. In turn, Fig. 19b shows the final result of the signal acquisition process corresponding to the three-phase voltages of 220/380 V electrical grid.

### 7.2 Calibration of the current measurement circuit

A calibration process similar to voltage sensors was carried out for current signal measurement and conditioning circuits. The results obtained from the calibration process are shown in Table 2.

**Table 2:** Characterisation of current sensors.

Sensor	Input current	Output voltage	Output/Input ratio
Sensor 1	10.40 A	620.1x$10^{-3}$ V	59.625x$10^{-3}$
Sensor 2	10.40 A	624.3x$10^{-3}$ V	60.02884615x$10^{-3}$
Sensor 3	10.40 A	626.1x$10^{-3}$ V	60.20192308x$10^{-3}$

**Fig. 20:**
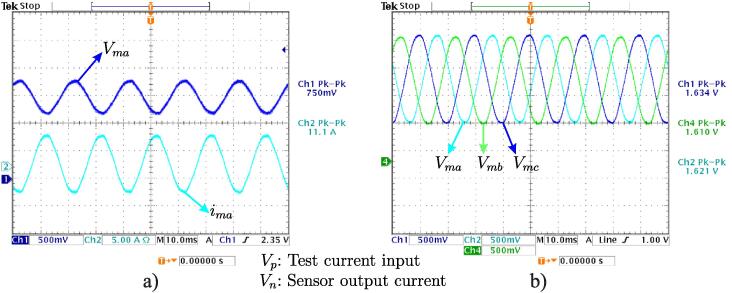
a) Test current input and current sensor output signals. b) Three-phase output signals measured with current sensors.

Fig. 20a shows input current signals used for the experiment and 3 V output signal obtained after the calibration process. In turn, Fig. 20b shows the final result of signal acquisition process corresponding to the three-phase test currents.

### 7.3 PWM pre-actuation circuit calibration

**Fig. 21:**
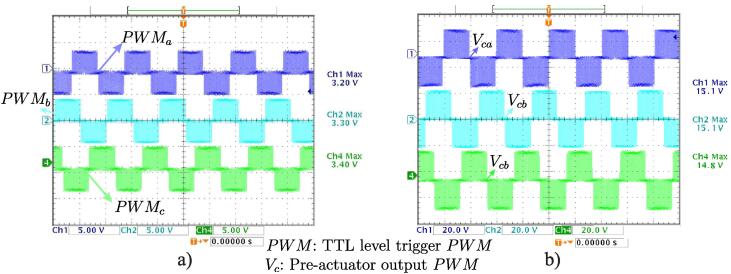
a) TTL level trigger PWM signals. b)15 V pre-actuator output PWM signals.

Regarding the pre-actuation block, the controller output voltage levels for PWM triggers produced by the DSP and the output levels obtained after the conditioning of the pre-actuation stage were measured. Fig. 21a shows the PWM trigger signals obtained at the output of the DSP controller. In turn, Fig. 21b denotes PWM output signals adapted to trigger logic levels for IGBTs of the VSI.

### 7.4 Experimental tests with the VSI converter

**Fig. 22:**
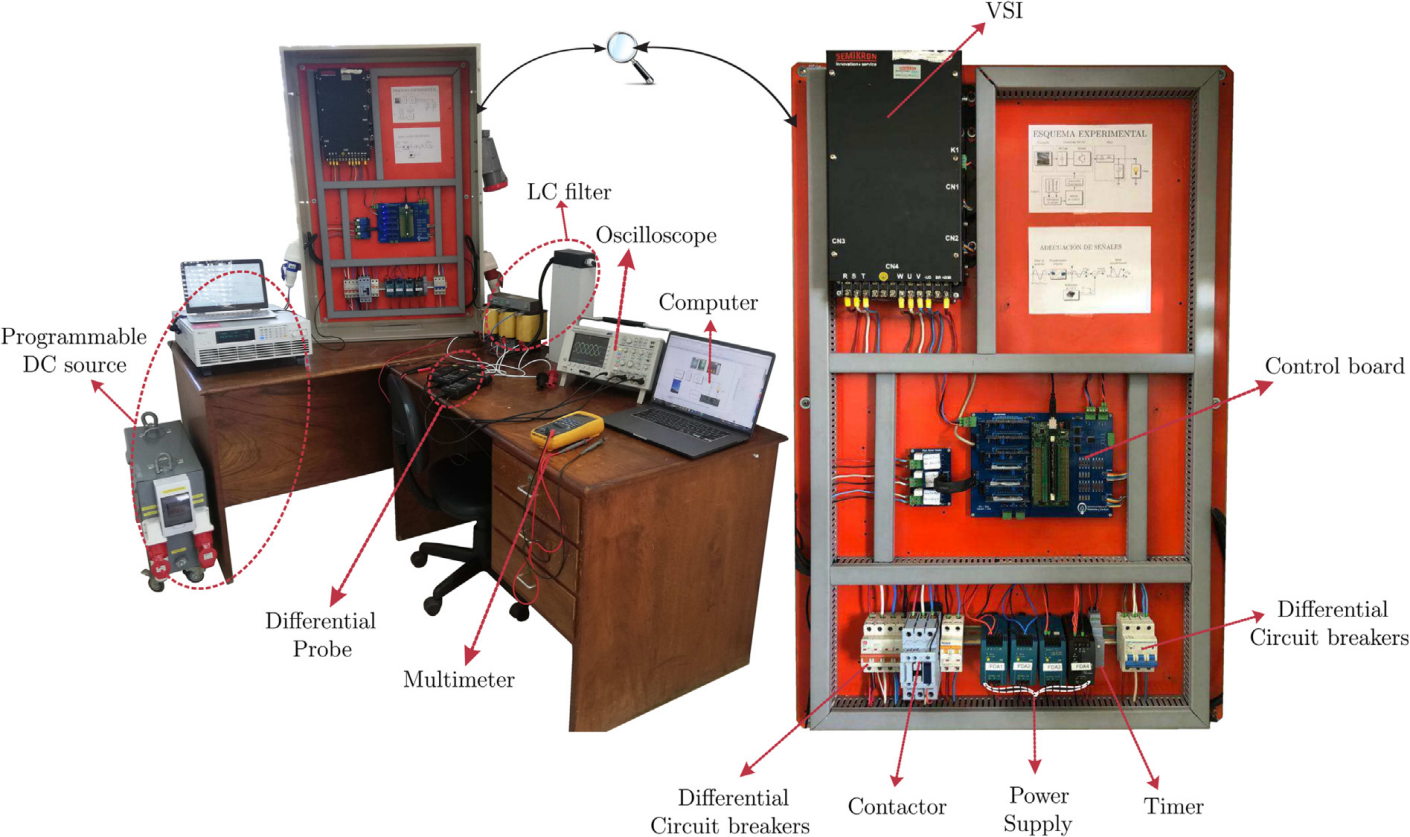
Photo of the experimental test bench based on the power conversion system and measurement equipment.

**Fig. 23:**
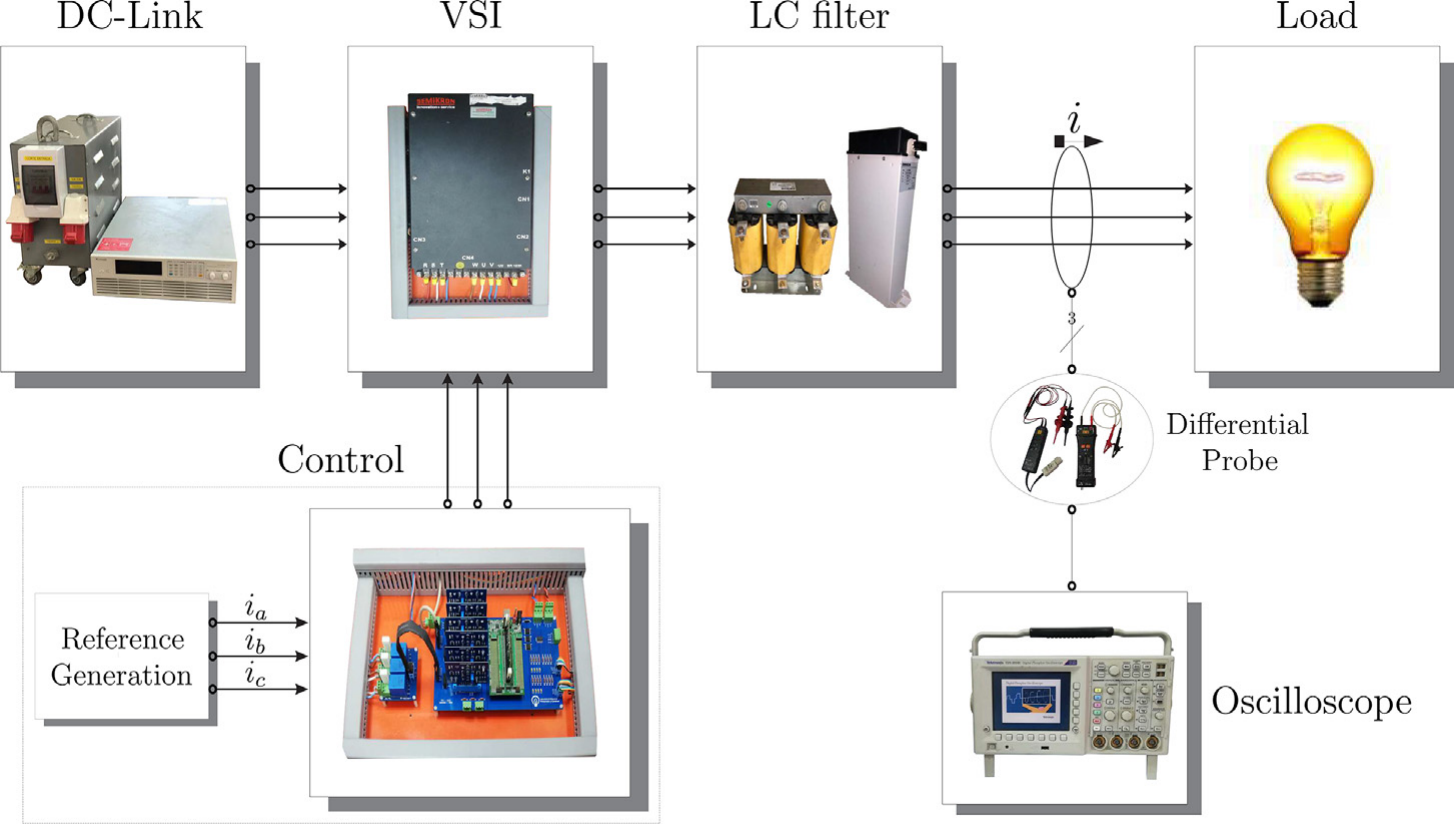
Experimental scheme of the power conversion system and measurement equipment.

Fig. 22 presents the experimental bench of the designed and implemented power electronics conversion system and measurement equipment used to validate the proposed design functionality. On the other hand, Fig. 23 shows the scheme of the implemented experimental platform. The reference signals are generated employing mathematical expressions that define the temporal behaviour of sinusoidal three-phase voltages, with a frequency equal to the electrical grid and an amplitude defined according to the delivered power into the system.

#### 7.4.1 Open-loop test

**Fig. 24:**
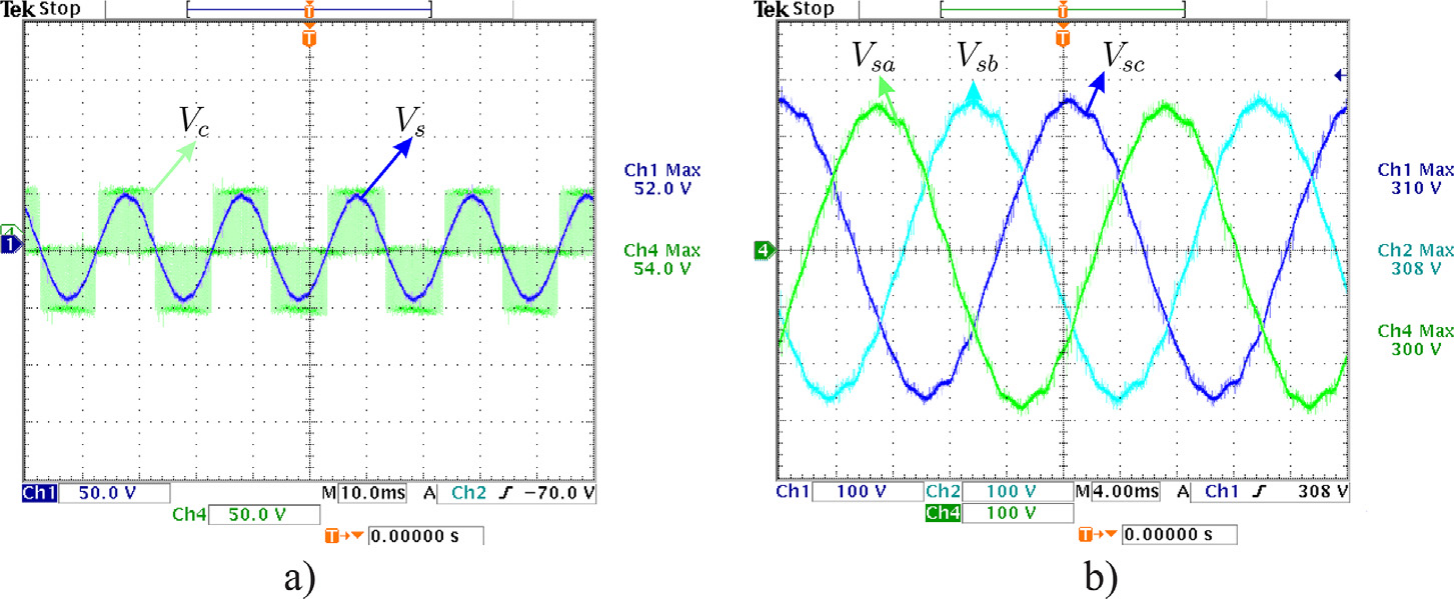
(a) PWM output signal of the converter. (b) Sinusoidal output signal obtained through LC filter.

Fig. 24a shows three-phase sinusoidal output voltage signals of the VSI. Both signals were obtained by measurements made with an oscilloscope. Output filtered through the LPF is shown in the same figure. By programming, a correct reference, appropriate PWM modulation, low-pass LC filtering, and three-phase sinusoidal voltage signals shifted 120° to each other is obtained. Once the correct operation of the system has been verified, experimental tests are continued with higher values of DC-link. Fig. 24b shows the system response when increasing the magnitude of the DC-Link, obtaining a sinusoidal signal with a maximum amplitude of 310 V and a frequency of 50 Hz.

#### 7.4.2 Closed-loop test

**Fig. 25:**
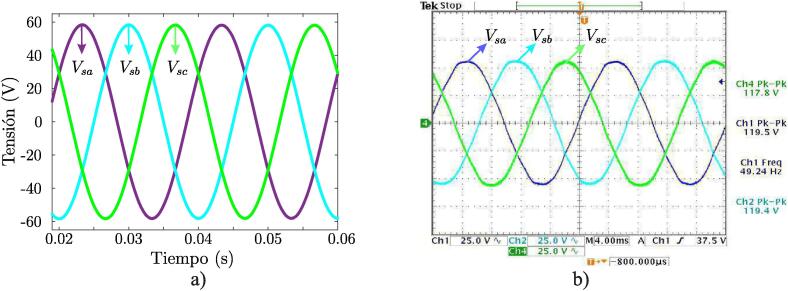
Closed loop voltages measured on the LC filter. a) Simulation results. b) Experimental results.

For closed-loop tests, the evaluated conditions in the simulations, using MATLAB/Simulink environment, were replicated, and subsequently, output voltage waveforms were obtained and captured with oscilloscope. The comparison between obtained waveforms through simulations and experimental measurements shows the correspondence between them and, therefore, the performance of the designed VSI. Fig. 25a shows output signals obtained through simulations and Fig. 25b shows output signals of the converter obtained through experimental tests.

#### 7.4.3 Total harmonic distortion analysis

**Fig. 26:**
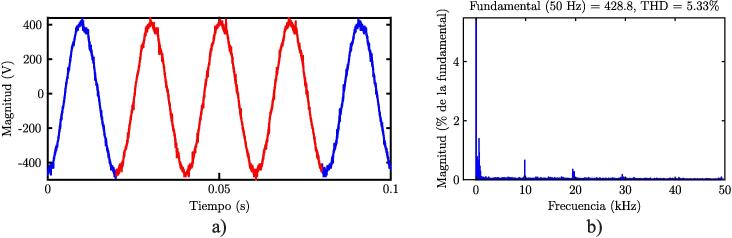
a) VSI converter output voltage. b) THD level measured experimentally in phase a of the system.

Finally, the quantification of Total Harmonic Distortion (THD) level is a parameter that indicates how much the signal’s harmonics cause voltage or current distortion [11]. This parameter constitutes a crucial figure of merit when analysing the efficiency of a power converter system. Fig. 26a,b show the analysis of THD level on the experimental platform for phase a of the system; a value of 5.3% was obtained. This result was obtained using the fast Fourier transformation, considered as a function of the oscilloscope. Mathematically speaking, THD is defined as:

(9) $$\mathrm{THD}(i_{s})=\sqrt[{}]{\frac{1}{i_{s1}^{2}}\overset{N}{\underset{j=2}{\sum }}{\left(i_{\mathrm{sj}}\right)}^{2}}$$

where $i_{s1}$ is the fundamental component of the measured currents and $i_{\mathrm{sj}}$ are the harmonic currents.

## 8 Conclusion

In this paper, the control system platform for a commercial VSI was designed to be a modular, scalable, multipurpose and open architecture. The electronic design was based on recommendations provided in the manufacturer technical data sheets, considering immunity criteria to noise, electrical insulation for protection, and an assembly that allows effective corrective maintenance.

Initially, experimental measurements were performed using reduced reference voltage levels to protect hardware’s integrity and evaluate the proposed design performance. Once this testing stage had been satisfactorily passed, tests were carried out at standard voltage levels for electrical networks.

Experimental measurements obtained with the designed voltage and current measurement and conditioning circuits have demonstrated that they meet the proposed objectives, presenting good linearity and reduced electrical noise levels within voltage levels admitted in the A/D converter modules of the DSP.

Regarding the levels of harmonic distortion in the output signal, a result of 5.3 % was obtained, which is within the expected range for this type of VSI and can be improved by implementing more elaborate control algorithms and modulation techniques.

## CRediT authorship contribution statement

**Hernan Lezcano:** Conceptualization, Methodology, Software, Visualization, Writing - original draft. **Jorge Rodas:** Funding acquisition, Project administration, Data curation, Writing - original draft. **Julio Pacher:** Conceptualization, Methodology, Supervision, Visualization. **Magno Ayala:** Writing - review & editing. **Carlos Romero:** Software, Validation.

## Declaration of Competing Interest

The authors declare that they have no known competing financial interests or personal relationships that could have appeared to influence the work reported in this paper.
